# Determinants of Cofactor Specificity for the Glucose-6-Phosphate Dehydrogenase from *Escherichia coli*: Simulation, Kinetics and Evolutionary Studies

**DOI:** 10.1371/journal.pone.0152403

**Published:** 2016-03-24

**Authors:** Matias Fuentealba, Rodrigo Muñoz, Pablo Maturana, Adriana Krapp, Ricardo Cabrera

**Affiliations:** 1 Laboratorio de Bioquímica y Biología Molecular, Facultad de Ciencias, Universidad de Chile, Santiago, Chile; 2 Instituto de Biología Molecular y Celular de Rosario, Facultad de Ciencias Bioquímicas y Farmacéuticas, Universidad Nacional de Rosario, Rosario, Argentina; Instituto de Tecnologica Química e Biológica, UNL, PORTUGAL

## Abstract

Glucose 6-Phosphate Dehydrogenases (G6PDHs) from different sources show varying specificities towards NAD^+^ and NADP^+^ as cofactors. However, it is not known to what extent structural determinants of cofactor preference are conserved in the G6PDH family. In this work, molecular simulations, kinetic characterization of site-directed mutants and phylogenetic analyses were used to study the structural basis for the strong preference towards NADP^+^ shown by the G6PDH from *Escherichia coli*. Molecular Dynamics trajectories of homology models showed a highly favorable binding energy for residues K18 and R50 when interacting with the 2'-phosphate of NADP^+^, but the same residues formed no observable interactions in the case of NAD^+^. Alanine mutants of both residues were kinetically characterized and analyzed with respect to the binding energy of the transition state, according to the *k*_cat_/K_M_ value determined for each cofactor. Whereas both residues contribute to the binding energy of NADP^+^, only R50 makes a contribution (about -1 kcal/mol) to NAD^+^ binding. In the absence of both positive charges the enzyme was unable to discriminate NADP^+^ from NAD^+^. Although kinetic data is sparse, the observed distribution of cofactor preferences within the phylogenetic tree is sufficient to rule out the possibility that the known NADP^+^-specific G6PDHs form a monophyletic group. While the β1-α1 loop shows no strict conservation of K18, (rather, S and T seem to be more frequent), in the case of the β2-α2 loop, different degrees of conservation are observed for R50. Noteworthy is the fact that a K18T mutant is indistinguishable from K18A in terms of cofactor preference. We conclude that the structural determinants for the strict discrimination against NAD^+^ in the case of the NADP^+^-specific enzymes have evolved independently through different means during the evolution of the G6PDH family. We further suggest that other regions in the cofactor binding pocket, besides the β1-α1 and β2-α2 loops, play a role in determining cofactor preference.

## Introduction

Substrate recognition depends on the interactions between the chemical groups of the ligand and the amino acid residues at the active site of the enzyme. For dehydrogenases, NAD^+^ and NADP^+^ are alternative cofactors whose differences in binding energy, determined by the presence of the 2’-phosphate, result in most of these enzymes being highly selective towards one of the cofactors. At their active sites, determinants of cofactor specificity often occur within conserved sequence motifs.

We have previously characterized the cofactor specificity of the glucose-6-phosphate dehydrogenase from *Escherichia coli* (*Ec*G6PDH), showing that the value of *k*_cat_/K_M_ for NADP^+^ is 410 times the value observed for NAD^+^ [1]. However, the first publication concerning the cofactor preference in different G6PDHs was provided by Levy [2] who compared the kinetic parameters for bacterial and eukaryotic enzymes. Five levels of cofactor specificity in G6PDHs were proposed, including NADP^+^- and NAD^+^-specific, NADP^+^- and NAD^+^-preferring, and dual enzymes. This initial classification was based on data of varying degrees of completeness and the distinctions were somewhat arbitrary. Later, Ragunathan and Levy in 1994 [3] pointed out that, in fact, no NAD^+^-specific G6PDHs had been characterized and suggested instead a classification including only three kinds of cofactor-binding sites: those accommodating only NADP^+^, those which accept either NAD^+^ or NADP^+^ under physiological conditions, and finally those binding either NAD^+^ or NADP^+^ *in vitro* but which are NAD^+^-preferring under physiological conditions. According to Cornish-Bowden [4], the preference for mutually exclusive alternative substrates (that recognize the same binding site) is better quantified by the ratio of *k*_cat_ to K_M_. However, in the case of G6PDHs the previous assessments have not been explicitly based on this consideration.

In terms of evolution, for some dehydrogenase families the dinucleotide specificity is a conserved trait associated with sequence motifs containing residues involved in NAD(P)^+^ recognition. For example, phylogenetic reconstruction based on the enzyme isocitrate dehydrogenase (IDH) (EC 1.1.1.42), shows that eukaryotic and prokaryotic members segregate into different groups characterized by their NAD^+^ or NADP^+^-specificity, and determined by motifs containing negative or positive residues, respectively [5]. This pattern led the authors to hypothesize that, deriving from a NAD^+^-based ancestor, the specificity for NADP^+^ evolved independently more than once in this family. In the case of the G6PDHs, phylogenetic analysis has shown that eukaryotes and bacteria diverged as independent groups with no signs of inter-domain horizontal transfer [6], but the phylogenetic distribution of cofactor specificity has yet to be addressed.

To date, the structures of G6PDH from *Homo sapiens*, *Leuconostoc mesenteroides* (*Lm*G6PDH), *Mycobacterium avium*, and *Trypanosoma cruzi* have been determined by X-ray crystallography [7–9]. The *Lm*G6PDH has been the most studied kinetically, with its specificity constant towards NAD^+^ being only 9.4 times that of NADP^+^ and, for this reason, has been classified as a dual enzyme [10]. Particularly, complexes of *Lm*G6PDH with NADP^+^ (PDB ID 1H9A), NAD^+^ (PDB ID 1H94), NADPH and D-glucose (PDB ID 1E7Y) have provided information about the residues modulating the preference for both dinucleotides, which have also been evaluated by site-directed mutations. We recently published the effect of the double mutant R46E-Q47E on the cofactor preference of *Lm*G6PDH [11]. In fact, we demonstrated that the mutant performed as a specific NADH-producer *in vivo*. To the best of our knowledge, this is the only member of the family for which site-directed mutagenesis has been employed specifically in order to identify the key residues involved in the binding of the dinucleotides. Unfortunately, information about structural determinants of a NADP^+^-specific G6PDH has not been reported.

In the case of *Lm*G6PDH, it has been estimated that the side chain of R46 contributes 3.1 kcal/mol to the binding energy of the transition state. This is based on the kinetic constants of the NADP^+^-dependent reaction [10]. Given that the *k*_cat_/K_M_ ratio is related to the activation energy, ΔG_ES_^‡^, it is possible to calculate the energy that a chemical group (a side chain in the protein, or the phosphate in the cofactor) contributes to the binding of the transition state [12]. For example, the energy contribution of specific amino acids has been assessed using this approach to determine their role in catalysis of tyrosyl-tRNA synthetase [13], and even in the folding of Barnase [14].

Molecular Dynamics (MD) simulations have been used as another tool to evaluate the specific role of certain amino acid residues in the binding of the cofactors NAD^+^ and NADP^+^, allowing for the quantification of the contributions coming from hydrogen bonds and other types of weak intermolecular interactions. In 2011, Zhao & Xiao compared the molecular basis underlying cofactor specificity in the wild type and mutant forms of human 3β-hydroxysteroid dehydrogenase by MD simulations [15]. The analysis of the complexes indicates that D35 and K36 are key residues for cofactor specificity in 3β-HSD1, which is in agreement with the results of mutagenesis studies. Likewise, for the L-lactate dehydrogenase from *Bacillus stearothermophilus*, an analysis of interatomic distances between cofactor and protein in MD simulations (including hydrogen bonds), has provided an explanation for the kinetic characteristics of a double mutant with altered NAD(P)^+^ specificity [16].

In this work, we modeled the structure of *Ec*G6PDH and identified, through MD simulations, residues K18 and R50 as determinants of its strong preference for NADP^+^. Following a double mutant cycle approach and using the cofactor specificity constants of wild type and mutant enzymes, we characterized the energetic interaction of these residues with the transition state of the reaction. Additionally, we searched for kinetic data for bacterial G6PDHs with different cofactor preferences, and analyzed their phylogenetic distribution. We found that the use of NADP^+^ as the preferred cofactor seems to be a common feature in part of the G6PDH family and it is associated with the conservation of R50. On the other hand, there is no conservation of K18 among the NADP^+^-preferring G6PDHs, instead the presence of a threonine residue is more frequent. For this reason, we analyzed the enzyme kinetics of the mutant K18T.

## Materials and Methods

### Structure Modeling of G6PDH

Homology models for the structure of *Ec*G6PDH were created using the crystallographic structure of *Lm*G6PDH in complex with NADP^+^ as the reference (PDB ID 1H9A). The alignment between the *Lm*G6PDH and *Ec*G6PDH sequences was obtained from the multiple sequences alignment generated as described in the section Phylogenetic Analysis of Bacterial G6PDHs. Ten three-dimensional models of *Ec*G6PDH complexed to NADP^+^ were generated using Modeller 9.10 [17]. The consensus secondary structure prediction by Jpred 3 [18] was used as a restraint during modeling. Subsequently, the models were ranked according to scoring functions implemented in Verify3D [19] and Prosa2003 [20]. The best model was used for loop refinement (https://salilab.org/modeller/manual/node35.html) to generate an additional ten models that were also analyzed and scored. From the best final model, the structure of the *Ec*G6PDH-NAD^+^ complex was obtained by removing the 2’-phosphate from the NADP^+^ complex. In order to take into account possible reorganization of the interactions in the binding site, 30,000 steps of conjugate gradient energy minimization were performed on the models.

### Molecular Dynamics

Molecular Dynamics simulations of the model complexes of *Ec*G6PDH with NADP^+^ or NAD^+^ were performed using NAMD (NAnoscale Molecular Dynamics) 2.8 [21] with the Amber99SB-ILDN force field [22] (which includes improved side-chain torsion potentials for the amino acids Isoleucine, Leucine, Aspartate and Asparagine) under explicit solvent, neutralized with Na^+^ ions in a box of TIP3P waters with a pad of 15 Å in all directions. The simulation was run using a time step of 1 fs, with a 12 Å cutoff for nonbonding interactions and a switching function from 10 Å for the Van der Waals and electrostatic terms. For the long-range interactions, the Particle Mesh Ewald method was applied. The simulation was carried out in the isothermal-isobaric (NPT) ensemble mode. The temperature was maintained constant through Langevin dynamics at 300 K, and the system was equilibrated until obtaining a constant RMSD for the backbone. Subsequently, the evolution of each system was simulated for 20 ns.

In the analysis stage, the hydrogen bonds were quantified by considering an angle of greater than 120° between donor, hydrogen and acceptor atoms, and a distance below 3.5 Å between donor and acceptor. The binding free energies per residue were calculated by using the MM/PBSA (Molecular Mechanics Poisson—Boltzmann Surface Area) method implemented in AMBER 11 [23]. Snapshots for the calculations were taken every 50 ps, resulting in a total of 400 snapshots per trajectory. The ionic strength in molarity units was set to 0.1. Other options were set as default. In order to visualize the free energy at the surface of the protein, the values obtained for the backbone and side chains in the previous analysis were used to replace the β-factor column of each residue in the PDB file of the model, using Tcl/Tk scripts executed in VMD 1.9 [24].

### DNA Techniques

The *zwf* gene in the plasmid pETG6PDH [1] was subcloned into the pET-TEV plasmid (derived from pET28-a by insertion of the TEV cleavage site and removal of the thrombin cleavage site and T7 tag). Primers were designed to introduce, by PCR amplification using pETG6PDH as the template, a site for *Nde*I in frame with the start codon of *zwf*, and the site for *Xho*I downstream of its stop codon. The PCR product and pET-TEV plasmid were digested with the respective enzymes and ligated. Colonies were evaluated for protein expression. The resulting plasmid was named pET-TEV-*Ec*G6PDH, and it expressed the coding sequence of *Ec*G6PDH preceded by a 21 amino acid sequence, including a 6xHis tag at the N-terminal end and a cleavage site for the TEV protease prior to the first Met of *Ec*G6PDH. After treatment with TEV protease, a dipeptide (Gly-His) is expected to remain before the first Met.

Site-directed mutagenesis was performed by PCR amplification of pET-TEV-*Ec*G6PDH, using mutagenic primers, according to the Genetailor^™^ system. To obtain K18A, the forward primer was 5'CTGGTCATTTTCGGCGCGGCAGGCGACCTTGCG (mutated codon underlined), and the reverse primer 5'CGCGCCGAAAATGACCAGGTCACAGGCCTG. To obtain R50A, the forward primer was 5'ATTATCGGCGTAGGGGCTGCTGACTGGGATAAAG (mutated codon underlined), and the reverse primer 5'CCCTACGCCGATAATCCGGGTGTCCG.

The double mutant K18A-R50A was prepared in consecutive steps using the same protocol. To ensure that no spurious mutations had been introduced, the entire gene was sequenced (Macrogen USA).

### Enzyme Isolation and Kinetic Assays

G6PDH K18A, R50A, K18A-R50A and K18T mutants were purified as described in [1]. The His-Tag was cut by TEV protease using a molar ratio of 1:50 (TEV:*Ec*G6PDH) at 25°C, overnight. Throughout the different stages of purification, the enzyme activity was followed by the increment in absorbance at 340 nm, using the following solution: Tris-HCl 10mM pH 8.2, 1mM MgCl_2_, NADP^+^ 1mM and G6P 2mM. Protein concentrations were determined using the Bradford reagent (BioRad), with bovine serum albumin as the standard.

Kinetic constants were determined at 25°C essentially as described by Olavarria *et al* 2012 [1]. Briefly, NAD(P)H formation was followed at 340 nm using a Synergy 2 spectrophotometer (BioTek) and 96-well plates, with a working volume of 300 μL. The substrates were added to the assay buffer immediately before loading the plate; reactions were triggered by addition of G6P. The assay was prepared using different concentrations of NADP^+^ or NAD^+^, at near-saturating concentrations of the co-substrate G6P (40 mM in the case of K18A and K18A/R50A, 50 mM for R50A; 15 mM for K18T; see Results). NADP^+^, NAD^+^ and G6P were purchased from Sigma-Aldrich (96.5% purity for NAD^+^ [N7004], 95% for NADP^+^ [N5755] and premium quality for G6P [G7879] according to the vendor) solutions were pH neutralized, and concentrations were enzymatically titred.

To obtain a good approximation of the initial rate, slopes were taken before 5% of initial substrate was consumed. Three independent measurements were taken and the standard deviation was calculated. Data was adjusted to the Michaelis-Menten equation to obtain K_M_ and *k*_cat_, by using SigmaPlot (Systat Software, San Jose, CA).

### Calculation of Interaction Energies

The binding energy in the transition state can be calculated from the specificity constant (*k*_cat_/K_M_) according to transition state theory [4]. This enables the calculation of differences in the transition binding energy between two different molecules interacting with the same enzyme, so long as they differ exclusively by a group P which is involved in binding interactions only and not in the chemical reaction itself. This can be done by calculating the ratio between the specificity constants for the smaller and the larger molecule. In our case this can be used to evaluate the contribution of the 2’-phosphate or the protein side chains to the binding energy of the transition state, by the following equation:

$$\Delta {\text{G}}_{\text{R}}=\text{RT ln}\left[{\left(k_{\text{cat}}/{\text{K}}_{\text{M}}\right)}^{\text{without P}}/{\left(k_{\text{cat}}/{\text{K}}_{\text{M}}\right)}^{\text{with P}}\right]$$ (1)

**Fig 1:**
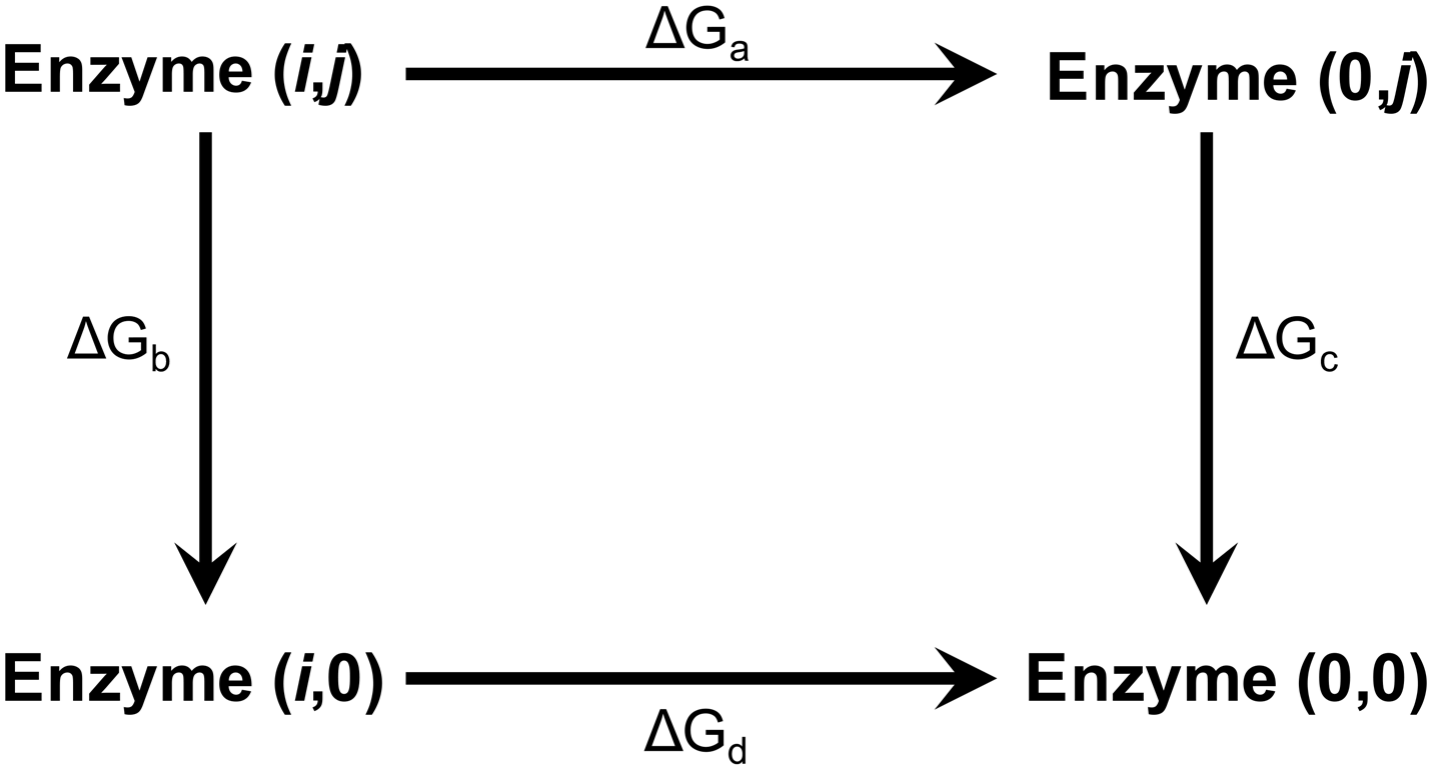
Double mutant cycle diagram. *i* and *j* represent the side chain residues in the wild type enzyme. The replacement by Ala is named “0”, so the wild type enzyme is denoted as (*i*,*j*) and the double Ala mutant as (0,0). The free energy change for the (*i*,*j*)→(0,*j*) transition, ΔG_a_, corresponds to the lack of the side chain *i* in the presence of the side chain *j*. ΔG_b_ stands for the opposite transition, (*i*,*j*)→(*i*,0). For the lack of the side chain *j* in absence of *i*, (0,*j*)→(0,0), and the lack of the side chain *i* in absence of *j*, (*i*,0)→(0,0), the energies are expressed as ΔG_c_ and ΔG_d_, respectively.

On the other hand, the energetic coupling between the K18 and R50 side chains was evaluated by the double mutant cycle approach, once again employing the specificity constants. In this case, Eq 1 can be adapted to assess the binding of one cofactor to wild type and mutant enzymes, where the mutant has a larger substrate binding pocket because it lacks a group (P) in its side chain (Fig 1). In this case, evidence of coupling comes from the fact that the free energy of the alanine-replacement of one side chain shows a different value when obtained in the presence or in the absence of the second side chain.

### Phylogenetic Analysis of Bacterial G6PDHs

In order to generate a phylogenetic tree of bacterial G6PDHs, we first searched the Swiss-Prot and TrEMBL (UniProtKB) databases for sequences of enzymes that have been kinetically characterized using NAD^+^ and NADP^+^ as cofactor. For 6 cases, we were able to find 1 G6PDH sequence among the bacterial species (or strains) for which kinetic characterization had been reported. For the remainder, either a specific strain was not found in UniprotKB (in other words, the sequence was annotated only up to the level of species), or several genes could be associated to the bacterial species or strain from which the G6PDH was characterized (authors did not report the specific isoform that was studied). We retrieved a total of 53 G6PDH sequences. After alignment of these sequences using ClustalX [25] we calculated a Neighbor Joining tree and focused on clusters of sequences from single organisms. Within each cluster, several sequences showed the same residue at positions 18 and 50 (*Ec*G6PDH numbering), and so only one representative was selected. After this step, 17 sequences remained, including representatives from the phyla Proteobacteria (α, β & γ), Firmicutes, Thermotogae and Aquificae. Four additional G6PDH sequences were included: those from *Mycobacterium avium* (Actinobacteria), whose structure is known (PDB ID 4LGV), from *Borreliaburgdorferi* (Spirochaetes), *Synechocisits* (Cyanobacteria) and *Chlamydophila pneumoniae* (Chlamydiae). The latter were included in order to enrich the number of analyzed phyla. In a preliminary tree we observed that G6PDH from *M*. *avium* coalesced with three proteobacterial sequences (*Gluconacetobacter hansenii b* and *c*, and *Pseudomonas fluorescens b*) to form a separate cluster. To better describe sequence conservation within this group, we searched the databases for sequences with less than 70% identity (to avoid redundancy). We ended up working with 31 bacterial G6PDH sequences (S1 Table). The structural alignment of the G6PDHs from *Leuconostoc mesenteroides* (*L*. *mesenteroides*) and *Mycobacterium avium* (*M*. *avium*) was used as an alignment profile in ClustalX to which the remainder of the sequences were subsequently aligned. This alignment was used to construct a Bayesian tree, using MrBayes [26] in a calculation of 5x10^6^ generations. Trees were sampled every 500 generations and the analysis was performed discarding the first 2500 trees.

## Results

### *In silico* study of NADP^+^-specificity in *Ec*G6PDH

In order to characterize differences between the interactions made by the nicotinamide dinucleotides with residues in the binding pocket of *Ec*G6PDH, we analyzed molecular dynamics simulations of complexes with these ligands.

**Fig 2:**
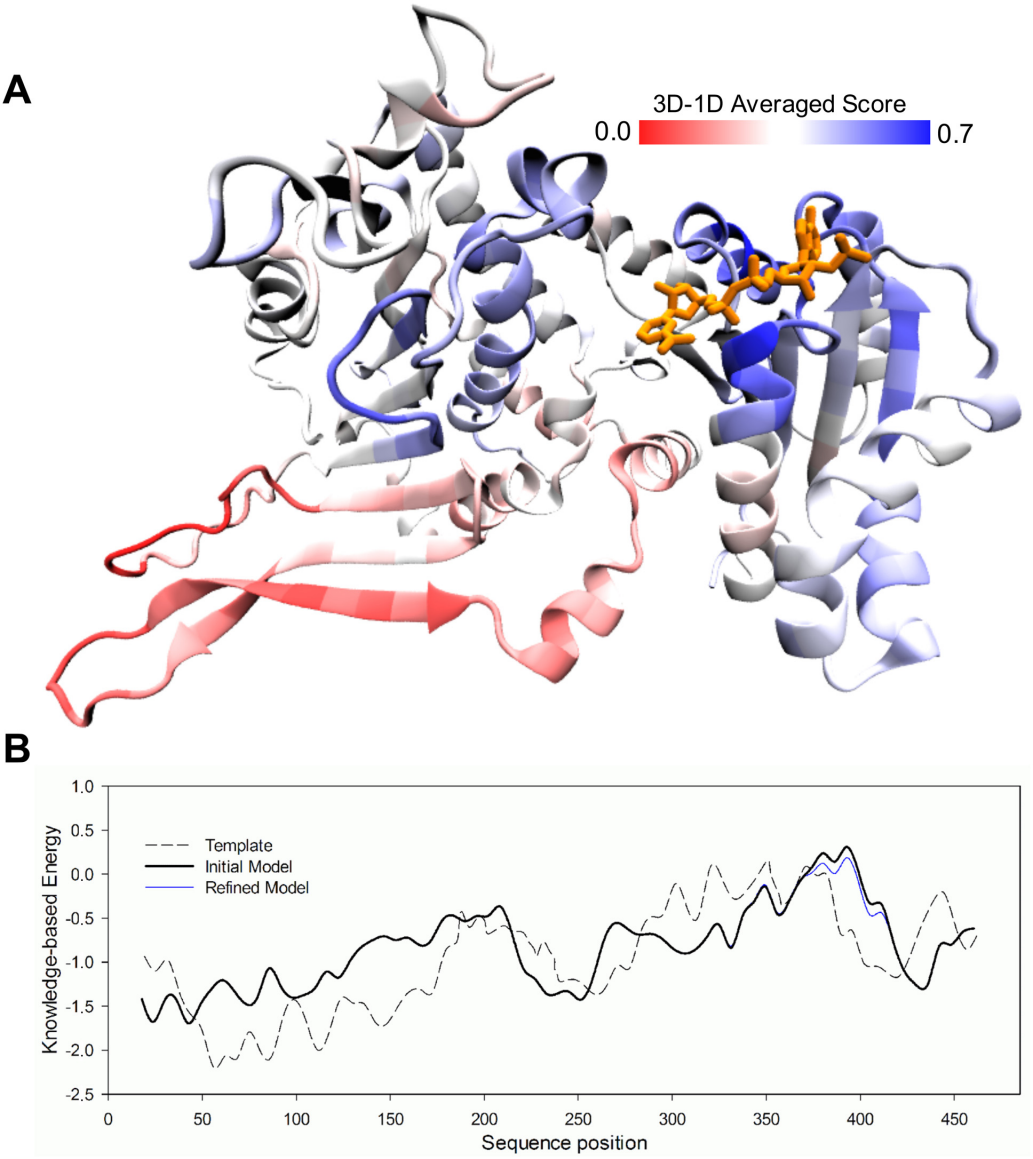
Homology model evaluation. (A) The protein structure of the refined model is displayed as a Cartoon representation and colored according to the 3D-1D Verify3D averaged score. Red and blue regions denote poor and good scores, respectively, as indicated by the scale at the top of the image. The dinucleotide is colored in orange. (B) Prosa2003 profile for the template (dashed line), initial model (black line) and refined model (blue line).

**Fig 3:**
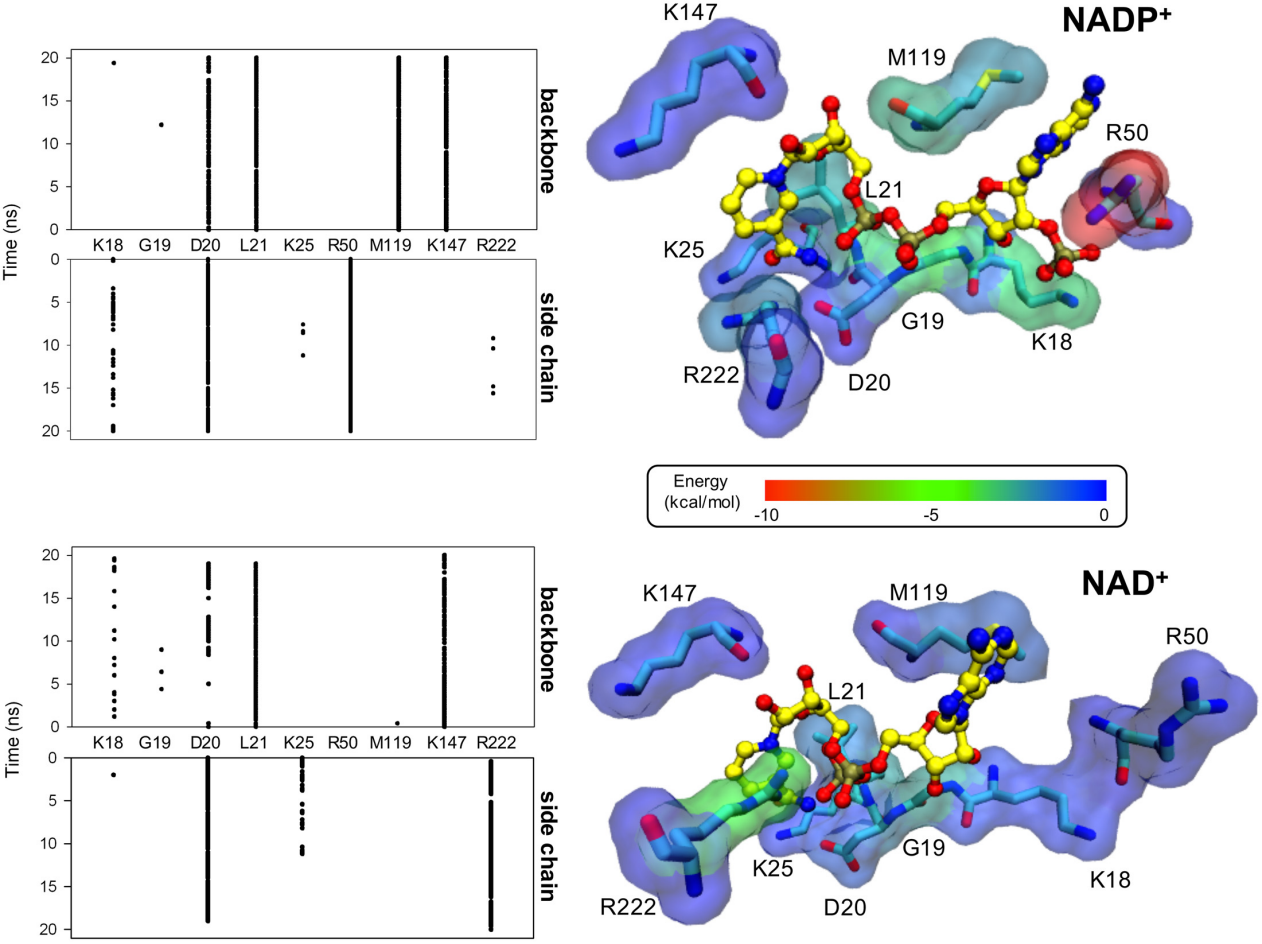
Interactions with cofactors at the nucleotide-binding site of the *Ec*G6PDH model. Left panel: Time course (sampled every 50 ps; see Materials and Methods for definition) of hydrogen bonds formed between NAD^+^ (lower) or NADP^+^ (upper) and the binding site residues, during the MD simulation. Interactions with the backbone and side chain atoms were counted separately. Right panel: Free energy contribution of the binding-site residues to cofactor binding, as calculated by MM/PBSA. The transparent surface representation is colored according to an energy scale, going from blue (no interaction) to red (the highest contribution).

Considering the availability of template structures, we generated molecular models of the enzyme complexed with NAD^+^ or NADP^+^, and evaluated the quality of these models by knowledge-based energy profiles with Verify3D [19] and Prosa2003 [20]. As depicted in Fig 2A, the cofactor binding site extends over the edge of the central β-sheet of the Rossmann domain. With regard to evaluation (Fig 2B), the regions around the β5-β6 loop, as well as β6, β8 and α9, within the C-terminal domain outside the vicinity of the cofactor-binding site, obtained low scores. According to the comparison with the crystallographic structure of the template, *Lm*G6PDH, these regions would be expected to be involved in the monomer-monomer interface [8]. Since we were able to improve the score of these regions through the standard loop refinement protocol from Modeller, we decided not to include the second monomer to complete the dimeric interface. In the final model structures, the averaged Verify3D scores were over 0.2 for 90% of the residues, and the best-evaluated residues were neighboring the cofactor binding site (blue regions, Fig 2A). After energy minimization, the modeled complexes were subjected to MD using NAMD. For each system, 20 ns of simulation were analyzed once stabilization of the root mean square deviation of the backbone with respect to the initial frame was observed. The role of the active site residues in NADP^+^ or NAD^+^ binding was assessed through the quantification of the hydrogen bonds and the binding free energy between the ligand and the enzyme (Fig 3).

The pocket around the 2’-phosphate of NADP^+^ is formed by two loops in the first half of the Rossmann domain (Fig 2A). There, the positive charges of K18 in the β1-α1 loop, and R50 in the β2-α2 loop, were observed to establish electrostatic interactions with the 2’-phosphate of NADP^+^. Fig 3 (upper left panel) shows that hydrogen bonds with these side chains can be observed for almost the entire simulation, with those from R50 being the more stable. Apart from these, D20 provides stable interactions through both its backbone (with the diphosphate moiety) and its side chain (with the nicotinamide group). In contrast, in the case of NAD^+^ the side chains of K18 and R50 play no role whatsoever in binding of the ribose-adenine moiety (Fig 3, lower left panel). As a consequence, this entire region of the cofactor tends to detach from its pocket, leaving only sparse interactions with the main chain of K18 and G19, and causing a destabilization of the hydrogen bond between the diphosphate moiety and the backbone of D20.

With respect to the ribose-nicotinamide half of the cofactor, where both dinucleotides are structurally equivalent, the hydrogen-bonding pattern was similar. For NAD^+^ and NADP^+^, the backbone of L21 and K147, as well as the side chain of D20, provided stable hydrogen bonds, helping to maintain the same orientation of their nicotinamide rings. Nevertheless, compared to NADP^+^, in the complex with NAD^+^ the interaction of the main chain of M119 with the hydroxyl groups of the ribose next to the nicotinamide, appeared to have been lost as a consequence of the partial detachment of the ribose-adenine half. Furthermore, K25 interacted with the nicotinamide group only during the first half of the simulation of the NAD^+^-complex. What is more, in the C-terminal domain the positive charge of R222 gained a stable interaction with the diphosphate moiety only in the complex with NAD^+^. If this interaction is indeed occurring in the complex with this cofactor under *in vitro* conditions, its importance does not seem to be significant for lowering its K_M_, which is almost three orders of magnitude higher than that of NADP^+^.

Since binding site residues could provide interactions other than hydrogen bonds, we decided to compare their contribution to the free energy of the protein-cofactor complex using MM/PBSA (Fig 3, right half). For the NADP^+^ complex, R50 plus K18 accounted for up to 60% of the total binding energy. Other residues contributed in the following order of importance: L21 > M119 > R222 > K25 > D20 > G19 > K147. On the contrary, the absence of the 2’-phosphate group in NAD^+^ precluded the interaction with both R50 and K18. As a consequence of the partial disjoining of the ribose-adenine moiety, G19, L21 and M119 also decrease their interaction energy, while R222 increased in importance by electrostatically interacting with the diphosphate.

Thus, our *in silico* analysis suggests that K18 and R50 from *Ec*G6PDH have a predominant function in positioning the 2’-phosphate of NADP^+^, thus giving the shape to the pocket and probably determining the preference of the site towards this cofactor. Also, in spite of having the same formal charge, their contribution seems not to be equivalent.

### The discrimination power of *Ec*G6PDH depends on the presence of K18 and R50

To evaluate the role of K18 and R50 on cofactor selectivity under *in vitro* conditions, we generated alanine mutants of these residues and determined their kinetic parameters in comparison with the wild type enzyme. We were thus able to determine the contribution of the side chains of K18 and R50 to the transition binding energy for NAD^+^ and NADP^+^.

**Fig 4:**
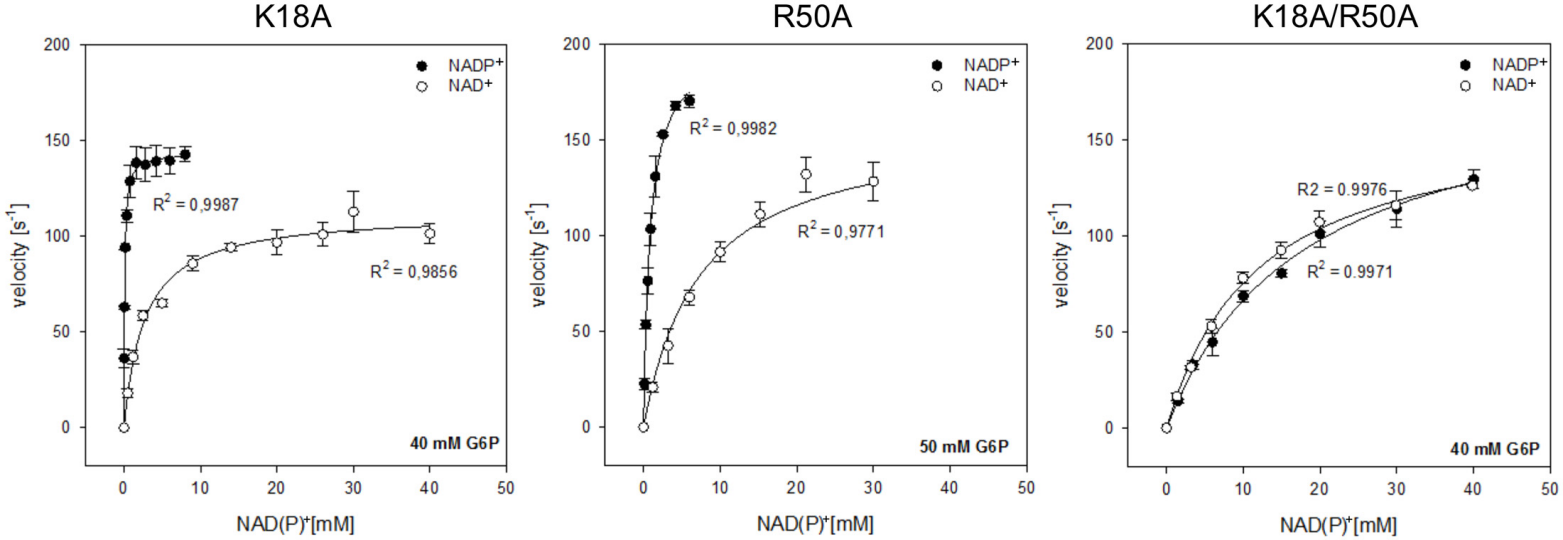
G6PDH activity of wild type and mutant enzymes as a function of NAD^+^ or NADP^+^ concentration. NADP^+^-dependent activity (black circles) and activity with NAD^+^ (white circles) was measured at nearly saturating co-substrate concentrations (bottom right corner). The data was fitted to the Michaelis-Menten equation and the Pearson correlation coefficient is indicated. The values for the kinetic parameters are given in Table 1.

Initial velocity of the oxidation of G6P was measured for K18A, R50A and the double mutant K18A-R50A, at different concentrations of NAD^+^ or NADP^+^ (Fig 4). For all the mutants, the activity was observed to depend hyperbolically on the concentration of the dinucleotide, and thus kinetic parameters were obtained by fitting to the Michaelis-Menten equation. Similar to wild type *Ec*G6PDH [1], in the case of single mutants, saturation is approached at lower concentrations of NADP^+^ than NAD^+^. However, Table 1 shows that the specificity constant (*k*_cat_/K_M_) decreased in the case of R50A more than for K18A, with a more pronounced effect in the NADP^+^-linked reaction (falling 46 and 16-fold, respectively) than in the NAD^+^-linked reaction (falling 6 and 1.5-fold, respectively). When using NADP^+^, this behavior is mainly accounted by the increase in K_M_ with respect to the wild type, since the changes in *k*_cat_ are rather modest for all the mutants. When using NAD^+^, the K_M_ value decreased with respect to the wild type by about 2-fold for K18A, whereas it increased by about 3 times and 2 times respectively for the R50A mutant and the double mutant. In terms of *k*_cat_, the NAD^+^-linked reaction decreased 2.6 times for K18A, and 1.7 times for both R50A and K18A-R50A. Most notably, the double mutant showed saturation curves with NAD^+^ and NADP^+^ that were virtually superimposable (Fig 4). For this mutant, the *k*_cat_/K_M_ for NADP^+^ fell more than 2000 times with respect to the wild type, almost exclusively due to a corresponding increase in K_M_ (Table 1). Additionally, we determined the effect of the mutations on the apparent kinetic parameters for G6P (S1 Fig). In the case of K18A for NAD^+^- and NADP^+^-dependent reactions, and R50A for the NADP^+^-dependent reaction, we could use cofactor concentrations at 10 times the determined K_M_. In the case of R50A for the NAD^+^-dependent reaction, and K18A-R50A for both the NADP^+^- and NAD^+^-dependent reaction, we used 30 mM (representing 2, 1.7 and 2.6 times the respective cofactor K_M_) to avoid absorbance artifacts in the measurements. Compared to wild type [1], the apparent K_M_ for G6P increases between 3.1 and 3.6 times when using NADP^+^ as cofactor, and between 1.4 to 3.2 times when using NAD^+^. With these results, we calculated that the apparent parameters for cofactors in Table 1 were determined at G6P concentrations over 90% saturation in all cases.

**Table 1:** Kinetic parameters of wild type and mutant enzymes.

	NADP^+^			NAD^+^		
*Ec*G6PDH	K_M_ (μM)	*k*_cat_ (s^-1^)	*k*_cat_/K_M_ (μM^-1^ s^-1^)	K_M_ (μM)	*k*_cat_ (s^-1^)	*k*_cat_/K_M_ (μM^-1^ s^-1^)
Wild type	7.5 ± 0.8	174 ± 2	23.2 ± 2.4	5090 ± 400	288 ± 5	0.06 ± 4x10^-3^
K18A	99 ± 3	143 ± 1	1.4 ± 0.04	2477 ± 260	109 ± 2	0.04 ± 4x10^-3^
R50A	382 ± 48	189 ± 5	0.5 ± 0.06	14662 ± 2176	171 ± 10	0.01 ± 1x10^-3^
K18A-R50A	17696 ± 1453	185 ± 7	0.01 ± 9x10^-4^	11736 ± 804	165 ± 4	0.01 ± 7x10^-4^

K_M_ and *k*_cat_ were determined from the hyperbolic fit shown in Fig 4.

The NADP^+^ preference of the mutants can be quantitatively expressed by dividing the specificity constant for NADP^+^ by that for NAD^+^. This ratio was used to estimate the binding energy of the 2’-phosphate group in NADP^+^ to the enzyme in the transition state. Table 2 shows that the absence of the Lys side chain causes a loss in NADP^+^ preference slightly higher than the absence of the Arg side chain (11-fold versus 8-fold). However, that difference is statistically insignificant when it is translated into energy. Thus, compared to wild type, the presence of only one positive charge at the 2’-phosphate pocket leads to a decrease from 3.4 to about 2 kcal/mol in the binding energy of this group to the transition state. According to the specificity constants of the double mutant, the absence of both positive charges renders the enzyme unable to discriminate between the dinucleotides. In other words, the energetic contribution of the 2’-phosphate to the binding of the transition state occurs through K18 and R50.

**Table 2:** Contribution of the 2’-phosphate of NADP^+^ to the binding energy of the transition state complex.

	Preference	Energetic contribution
*Ec*G6PDH	(*k*_cat_/K_M_)^NADP+^/ (*k*_cat_/K_M_)^NAD+^	ΔG^≠^ (kcal/mol)
Wild type	386 ± 51	-3.4 ± 0.08
K18A	35 ± 4	-2 ± 0.06
R50A	50 ± 10	-2.1 ± 0.1
K18A-R50A	1 ± 0.1	0 ± 0.05

The quotient between the specificity constants for NADP^+^ and NAD^+^ was used to calculate the energetic contribution, according to the Eq 1.

**Fig 5:**
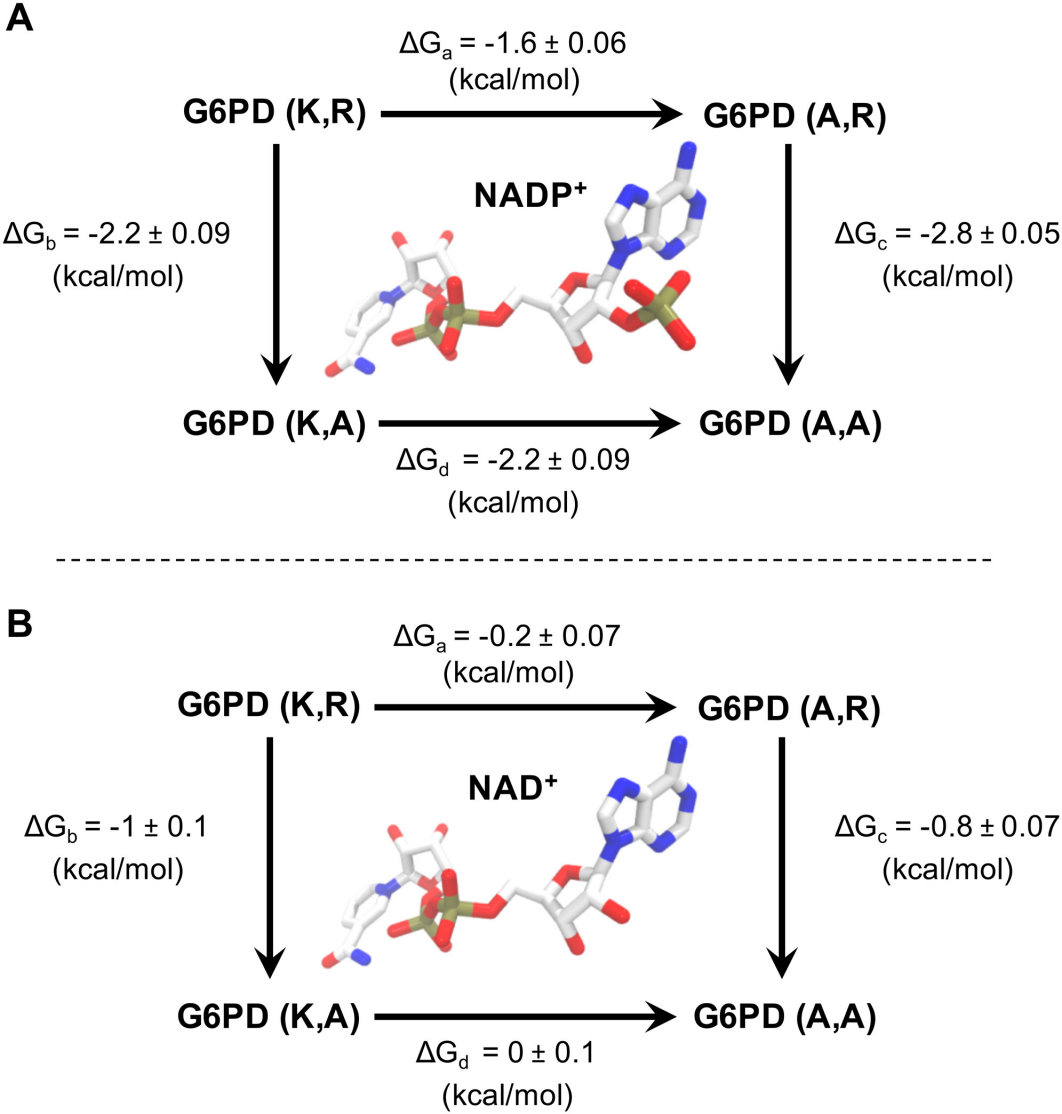
Energetic coupling between K18 and R50 of *Ec*G6PDH. The binding free energy changes of the transition state complex with NADP^+^ (A) or NAD^+^ (B) is analyzed as a double mutant cycle. In the case of the NADP^+^ complex, for each residue its contributed energy changes by about 0.6 kcal/mol depending on the presence or absence of the second positively charged residue. In the case of NAD^+^ binding, the coupling between these residues is less significant.

Taking into account that the binding energy of the phosphate group observed when two positive charges are present (wild type) does not double the value obtained when there is only one charge (any of the single mutants), we decided to assess the individual contribution of each side chain by applying the scheme of a double-alanine mutant cycle. Fig 5A shows that for the NADP^+^-linked reaction, removal of the K18 side chain causes a decrease of 1.6 kcal/mol in the binding energy of the transition state when R50 is present in the active site, but this increases to 2.2 kcal/mol when it is absent. In turn, the removal of R50 causes a diminution of 2.2 kcal/mol in the presence of K18, but in its absence the observed loss is 2.8 kcal/mol. This behavior indicates a negative energetic coupling between these side chains with regard to 2’-phosphate binding. In particular, each lowers by 0.6 kcal/mol the energetic contribution of the other. Conversely, Fig 5B shows for the NAD^+^-linked reaction that the removal of K18 caused a 0.2 kcal/mol loss in the transition-state binding energy in the presence of R50 but no loss at all in its absence. Likewise, the energetic contribution of R50 increased from -0.8 to -1 kcal/mol when Lys was present at position 18. Therefore, in the absence of the 2’-phosphate group in the cofactor, K18 and R50 showed a positive coupling, each increasing by 0.2 kcal/mol the contribution of the other to the binding energy in the transition state.

### Evolution of cofactor preference in bacterial G6PDHs

Our kinetic analysis using wild type and mutant *Ec*G6PDHs demonstrated that the presence of the side chains of K18 and R50 is crucial to confer discriminatory power and high catalytic efficiency towards NADP^+^. Consequently, we wanted to analyze the conservation of these residues among other G6PDHs, specifically in regard to the evolution of cofactor specificity in this family.

With this purpose in mind, we first searched for bacterial G6PDHs whose kinetic parameters have been characterized for both NADP^+^ and NAD^+^, so their preference could be quantitatively assigned. Table 3 summarizes the kinetic parameters of 15 bacterial G6PDHs. Since the cofactor preference could be defined in terms of the quotient between the respective specificity constants [4], we decided to classify three different levels of cofactor discrimination: when the quotient was below ten-fold, the enzymes were regarded as dual; the term “preferring” was used for quotients between 10 and 100-fold, and the category “specific” when the quotient was above 100-fold. According to our criterion, the G6PDHs from *Gluconacetobacter hansenii* (*G*. *hansenii*), *Pseudomonas fluorescens* (*P*.*fluorescens*), *Azotobacter vinelandii* (*A*.*vinelandii*), *Zymomonas mobilis* (*Z*.*mobilis*), *Aquifexaeolicus* and *L*. *mesenteroides* can be considered dual. While the G6PDH from *Gluconobacter oxydans* (*G*.*oxydans*) belongs to the NADP^+^-preferring category, those from *E*. *coli* and *Thermotoga maritima* (*T*.*maritima*) were deemed to be NADP^+^-specific enzymes. Furthermore, the data in Table 3 suggest that for G6PDHs the cofactor preference is mainly dictated by the differences between K_M_ rather than *k*_cat_ values. Thus, we used the ratio between the K_M_ of NAD^+^ and NADP^+^ as a proxy for cofactor preference in those cases for which the specificity constant was not reported. Following this reasoning, the G6PDHs from *Streptomyces aureofaciens* (*S*. *aureofaciens*) and *Pseudomonas aeruginosa* were classified as dual, the enzymes from *Burkholderia multivorans* (*B*.*multivorans*), *Methylomonas* and *Burkholderia cepacia* as NADP^+^-preferring, and that from *Bacillus licheniformis* as NADP^+^-specific. Except for *Burkholderia cepacia*, *Methylomonas* and *Streptomyces*, we were able to find in UniprotKB one or more G6PDH sequences for each microorganism.

**Table 3:** Kinetic parameters of bacterial G6PDHs.

	NADP^+^			NAD^+^			Specificity quotients				
Organism	K_M_ (μM)	*k*_cat_ (s^-1^)	*k*_cat_/K_M_ (μM^-1^ s^-1^)	K_M_ (μM)	*k*_cat_ (s^-1^)	*k*_cat_/K_M_ (μM^-1^ s^-1^)	(*k*_cat_/K_M_)^NADP+^/ (k_cat_/K_M_)^NAD+^	(K_M_)^NAD+^/ (K_M_)^NADP+^	Reference	Sequences in the UniprotKB Database	Sequences in phylogenetic tree
*Gluconacetobacter hansenii*	340	1288	4	104	2133	21	0.2	0.3	[3]	3	3
*Pseudomonas fluorescens*	360	1117	3	150	1383	9	0.3	0.4	[27]	33	2
*Azotobacter vinelandii*	50	37	0.7	220	92	0.4	1.8	4.4	[28]	4	2
*Zymomonas mobilis*	40	338	8	210	589	3	3	5.3	[29]	1	1
*Streptomyces aureofaciens*	75	-		140	-		-	1.9	[30]	0	0
*Aquifex aeolicus (70°C)*	161	894	6	2096	2012	1	6	13	[31]	1	1
*Leuconostoc mesenteroides*	8	522	65	162	1125	7	9.4	20	[10]	1	1
*Burkholderia multivorans*	40	-	-	500	-	-	-	13	[32]	2	2
*Pseudomonas aeruginosa*	57	-	-	333	-	-	-	6	[33]	1	1
*Methylomonas*	14	-	-	200	-	-	-	14	[34]	0	0
*Gluconobacter oxydans*	26	43	1.6	740	43	0.1	28	28	[35]	1	1
*Burkholderia cepacia*	20	-	-	1800	-	-	-	90	[36]	0	0
*Escherichia coli*	8	178	22	5224	280	0.1	415	653	This study	1	1
*Bacillus licheniformis*	5	-	-	3000	-	-	-	600	[37]	4	1
*Thermotoga maritima*	40	35000	875	12000	11000	0.9	955	300	[38]	1	1

Bacterial G6PDHs with characterized K_M_ and *k*_cat_ for NAD^+^ or NADP^+^ found in literature. The cofactor preference is inferred from the quotients of the specificity constants (NADP^+^ over NAD) or the K_M_ (NAD^+^ over NADP^+^), as described in Results. G6PDH sequences were searched in UniprotKB for the reported species or strain and the total number of sequences found and the number selected for phylogenetic analysis is indicated. The sequence identifiers are listed in S1 Table. Almost all K_M_ and *k*_cat_ values were apparent values with the exception of those from Methylomonas, which were calculated at four different G6P concentrations and five concentrations of NAD(P)^+^.

**Fig 6:**
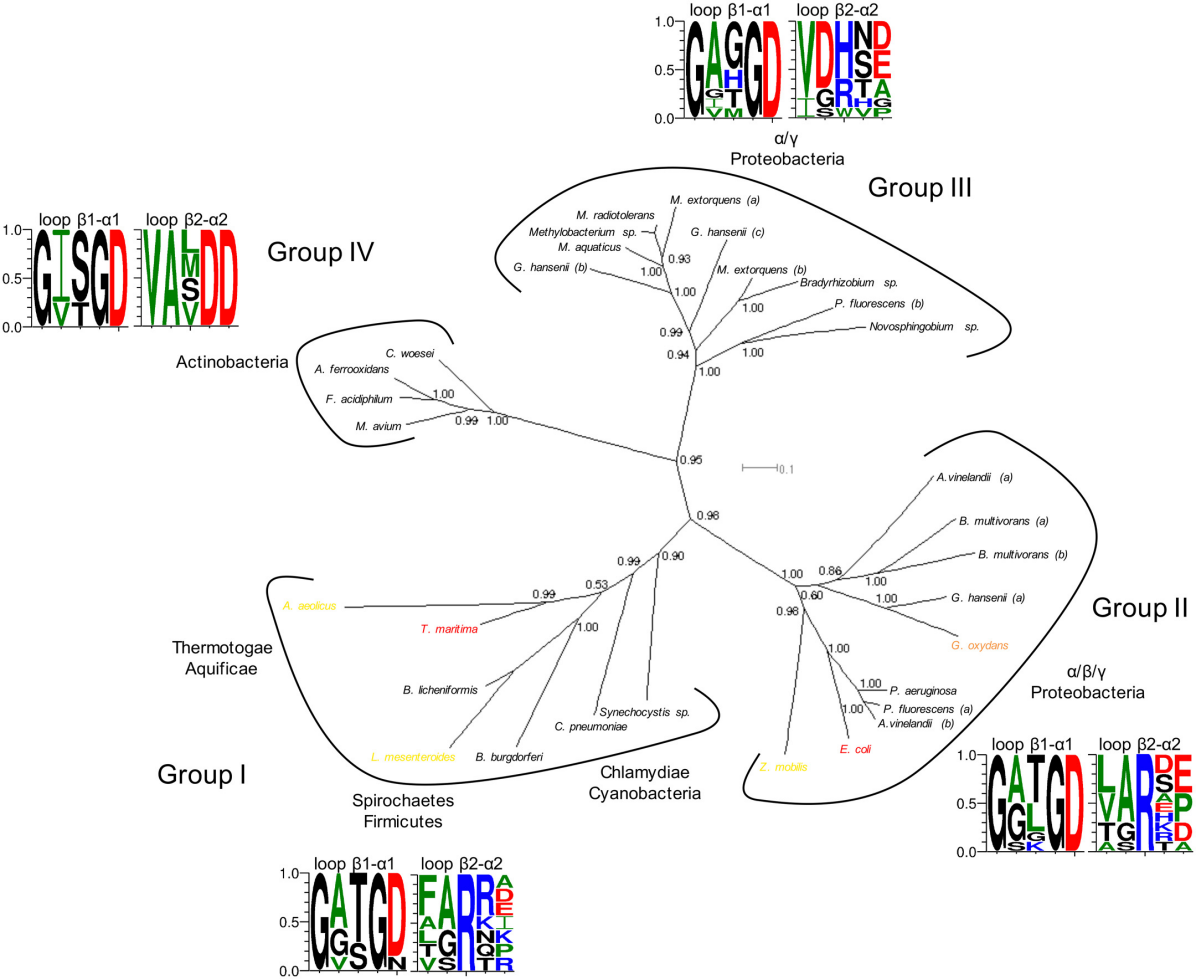
Evolution of the bacterial G6PDHs with regard to cofactor specificity and sequence motifs. Bayesian tree built from a structurally guided multiple sequence alignment. The species for which kinetic data are available are colored according to their cofactor specificity: red, NADP^+^ specific; orange, NADP^+^-preferring; yellow, dual enzymes. The main clusters are surrounded by a black line and for each one the phylum of the species involved are named. In addition, the sequence logos in terms of probabilities for the sequence motifs aligning with the structural loops containing K18 and R50 (loops β1-α1 and β2-α2) of *Ec*G6PDH are displayed. The posterior probabilities are shown for each node.

Fig 6 shows a Bayesian phylogenetic tree built for 31 G6PDHs, highlighting those with assigned cofactor preference. Four main groups compose the tree. Group I contains most of the bacterial lineages (Thermotogae, Aquificae, Spirochaetes, Firmicutes, Chlamydiae and Cyanobacteria). Proteobacteria cluster into two distinct branches, one with α-,β- and γ- representatives (group II) and the other with only α- and γ- members (group III). Interestingly, group III is more closely related to the actinobacterial branch (group IV) than to the proteobacterial group II. Paralogous G6PDHs are found only among Proteobacteria. G6PDH variants from *A*.*vinelandii* and *B*.*multivorans*, belong to group II, while those from *M*. *extorquens* are found within group III. In the cases of *P*. *fluorescens* and *G*. *hansenii*, variants distribute between groups II and III.

More remarkably, all the G6PDHs that could be unequivocally assigned to a given cofactor preference are scattered within groups I and II, with preferences going from dual to NADP^+^-specific in each group. Although the data on cofactor preference of G6PDH is sparse, the observed pattern is sufficient to reject the possibility that G6PDHs with the same cofactor preference conform to monophyletic taxons.

The sequence pattern of the β1-α1 loop in the different groups indicates that there is no conservation of K18. Indeed, this position shows a high frequency of Thr in groups I and II, or Ser in group IV. In the case of the β2-α2 loop, the R50 residue is completely conserved only in groups I and II, but for group III, the conservation of Arg decreased in favor of His, and Asp is frequently observed in the previous position. In the actinobacteria there is no conservation in the position equivalent to R50, but two conserved aspartic acids appear in the following two positions.

**Fig 7:**
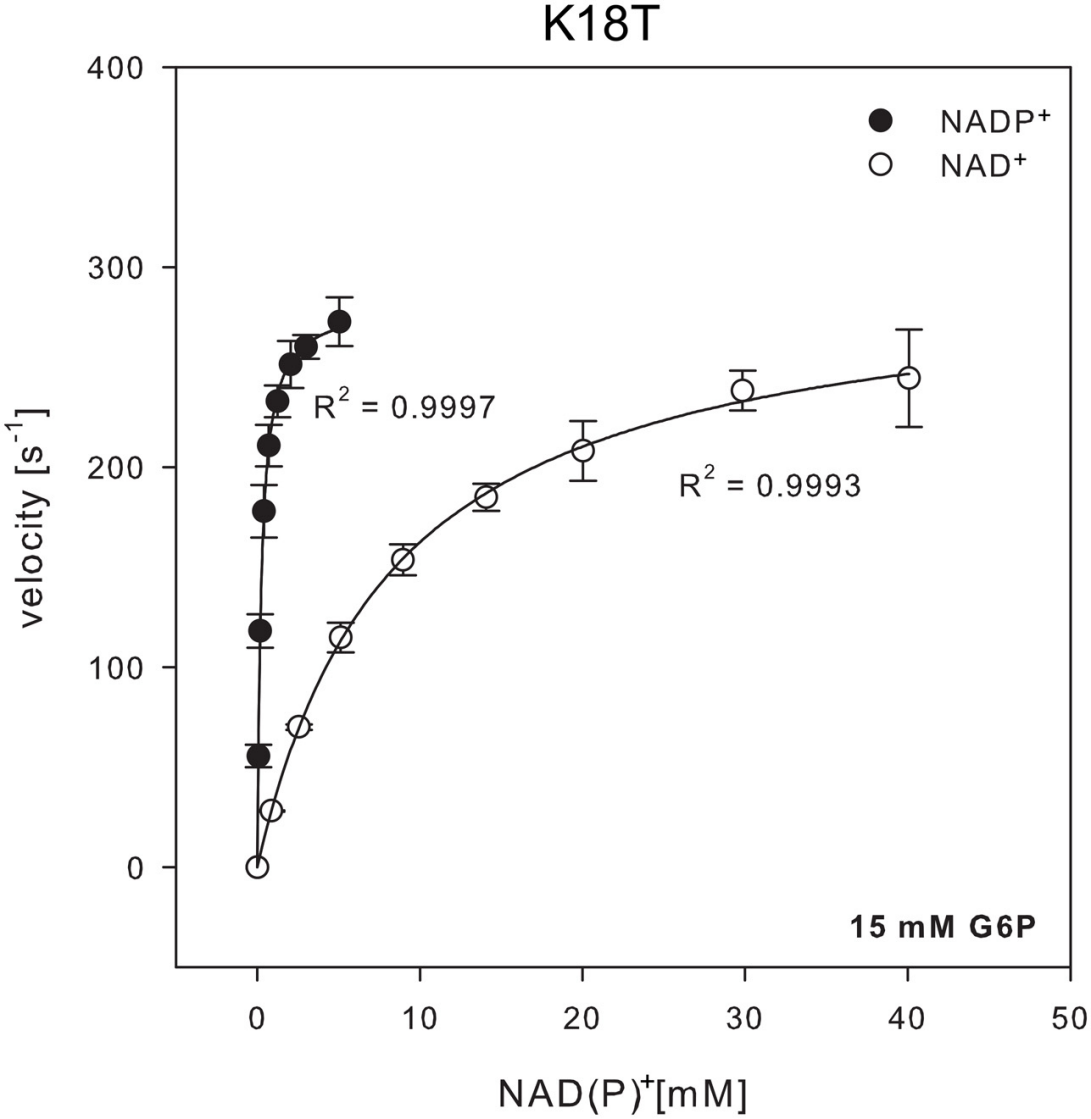
G6PDH activity of K18T mutant enzyme as a function of NAD^+^ or NADP^+^ concentration. NADP^+^-dependent activity (black circles) and activity with NAD^+^ (white circles) was measured at nearly saturating co-substrate concentrations (bottom right corner). The data was fitted to the Michaelis-Menten equation and the Pearson correlation coefficient is indicated. The values for the kinetic parameters are given in Table 4.

Since Thr at position 18 is present in several of the dual G6PDHs, particularly in *L*. *mesenteroides* where its role in NAD^+^ binding has been demonstrated [39], we generated the K18T mutant of *Ec*G6PDH in order to evaluate if its kinetic performance with NAD^+^ is improved. Fig 7, illustrates the velocities of the mutant enzyme at different NAD(P)^+^ concentrations and Table 4 indicates its kinetic parameters. Compared with the wild type, the specificity constants for both cofactors decreased in the mutant enzyme with a more pronounced effect in the NADP^+^-linked reaction over the NAD^+^-linked reaction (falling 20-fold and 2-fold, respectively). Not only did the specificity constant fail to increase for the NAD^+^-linked reaction, but also the preference for NADP^+^ decreased to the level of the K18A mutant. As described for our Ala mutants we determined the effect of K18T mutation on the apparent kinetic parameters of G6P (S1 Fig). In the case of the NADP^+^-dependent reaction we used a concentration of 10 times the determined cofactor K_M_, for the NAD^+^-dependent reaction we used 30 mM (representing 3.6 times the cofactor K_M_) to avoid absorbance artifacts. Compared to wild type [1], the apparent K_M_ for G6P increased 3.7 times in the presence of NADP^+^, but reduced to 80% when using NAD^+^ as cofactor. With these values, we calculated that the apparent parameters for cofactors were determined at 96 and 94% saturation G6P, respectively.

**Table 4:** Kinetic parameters of the K18T mutant enzyme.

EcG6PDH K18T	K_M_ (μM)	*k*_cat_ (s^-1^)	*k*_cat_/K_M_ (μM^-1^ s^-1^)
NADP^+^	243 ± 5.3	282 ± 1	1.1 ± 0.03
NAD^+^	8352 ± 307	298 ± 4	0.03 ± 1x10^-3^

K_M_ and *k*_cat_ were determined from the hyperbolic fit shown in Fig 7.

## Discussion

In this work the determinants of cofactor preference for G6PDH from *Escherichia coli* were studied by molecular simulation, kinetic characterization of site directed mutants in an alanine cycle approach, and by phylogenetic analysis. We have demonstrated that the positive charges at positions 18 and 50 are the main features responsible for the marked selectivity which the enzyme exhibits towards NADP^+^.

The roles of K18 and R50 could be distinguished. The guanidinium group of R50 interacts with the 2’-phosphate by electrostatic complementarity as well as stacking against one face of its aromatic ring of the adenine, where it is aided by M119 on the opposite side. K18, on the other hand, appears to be specifically involved in helping to neutralize the negative charge of the 2’-phosphate group. What is more, the quantification of hydrogen bonds showed that the side chain of K18 interacts with the ligand at a lower frequency compared with R50 (Fig 3). The kinetic experiments indicated that the binding energy of the 2’-phosphate in the transition state was about 2 kcal/mol when either of the two positively charged residues was present, but changed to 3.4 kcal/mol in the presence of both. Interestingly, this lack of additivity appears to be caused by an antagonistic energetic coupling of 0.6 kcal/mol between K18 and R50 when they bind the 2’-phosphate, as observed in the double mutant cycle (Fig 5). Thus, in the presence of one another, K18 and R50 do not achieve their individual maximum potential interaction with the ligand.

In the complex with NAD^+^, K18 and R50 have a diminished contribution to the binding of the ribose-adenine half of the cofactor. In fact, the most striking feature of our simulation was the partial detachment of the adenosine region, due to the absence of interactions with the side chains of K18 and R50 (Fig 3). Even so, the double alanine mutant cycle showed that R50 provides between 0.8 and 1 kcal/mol to the binding energy of the transition state, providing some evidence that some interaction was maintained, probably through π-cation non-covalent bonding with the adenine ring. Moreover, K18 showed a binding energy of 0.2 kcal/mol to the transition state with NAD^+^ only in the presence of R50 and not in its absence (Fig 5). These discrepancies with our simulations could be due to the inefficacy of the Molecular Mechanics force fields to represent the π-stacking interaction between the adenine and Arg side chain [40].

Differential interactions between NAD^+^ and NADP^+^ protein complexes have been analyzed through MD simulations in other systems. For example, in the family of short-chain dehydrogenases [41], the recognition of the adenine ribose from NAD^+^ is dominated by the presence of an Asp and the recognition of the 2’-phosphate in the case of NADP^+^ complexes is frequently performed by an Arg. However, this analysis involved considerably shorter simulations (0.5 ps), not intended to evaluate the stability of protein-ligand binding. In addition, we previously reported MD simulations of the dual *Lm*G6PDH with NAD^+^ and NADP^+^, based on its crystallographic complexes [11]. In this enzyme, the R46 residue is homologous to R50 of *Ec*G6PDH (Fig 6) and has been shown to perform an equivalent function in the interaction with the 2’-phosphate of NADP^+^ [8]. Furthermore, T14 in *Lm*G6PDH aligns with K18 of the *E*. *coli* enzyme and is important for interacting with the 2’-phosphate and the ribose-3’-hydroxyl group of NADP^+^. Interestingly, in opposition to *Ec*G6PDH, the simulations of the *Lm*G6PDH-NAD^+^ complex showed that the ribose-adenine region was stable in its pocket, mainly because of stable hydrogen bonding with the main chain atoms of T14 and R46, together with a stabilizing interaction with the side chains of Q47 and T14 [11].

Whereas the phylogeny of G6PDHs has been previously addressed [6, 42, 43], this is the first report to map cofactor specificities onto the evolutionary tree. In the G6PDH family, eukaryotes and bacteria constitute clearly separated clades [6], and no homologues have been found in the Archaea domain [43]. Also, it was observed that Proteobacteria representatives do not branch as a single clade [6, 42]. Besides, Lee and Coté (2006) used *zwf* and other housekeeping genes to trace phylogenetic relationships among γ-Proteobacteria, but they did not show any evidence of paralogy (*A vinelandii*, and *P*. *fluorescens* were not included in their analysis). As in our case, Canback *et al* [6] found Actinobacteria (*Mycobacterium tuberculosis*) associated to an α-γ Proteobacterial clade. Our evidence supports the idea that duplication and horizontal transfer events involving γ-Proteobacteria have occurred in the G6PDH family. When considering cofactor preference, we see that dual, NADP^+^-preferring and NADP^+^-specific enzymes are scattered among groups I and II, but it is not possible an unequivocal assignment of cofactor specificities in groups III and IV. For example, the G6PDH variants of *P*. *fluorescens* and *G*. *hansenii* are present in both proteobacterial groups II and III, and also two G6PDHs activities have been reported in each organism [27, 3]: One dual (Table 3) and one NADP^+^-preferring in the case of *P*. *fluorescens* (its reaction rate with NAD^+^ is 55% of the rate with NADP^+^); and one dual (Table 3) and one NADP^+^-specific in the case of *G*. *hansenii*. However, it is not known which activity corresponds to which sequence. With regard to those species for which we could not find a sequence in the database: (i) *Burkholderia cepacia*, characterized as NADP^+^-preferring, it is a β-proteobacteria and so we would expect to find it within group II; (ii) the dual G6PDH of *Methylomonas*, which is a γ-proteobacteria, could in principle be associated with clusters II or III; and (iii) the dual G6PDH of the actinobacteria *Streptomyces*, could be taken to sustain that the two Asp from the β2-α2 loop of members of this clade, do not confer discrimination against NADP^+^. However, neither the sequence nor the *k*_cat_ for cofactors are known for this enzyme. Groups III and IV must await further studies focused on cofactor preference.

Identification of the cofactor specificity determinants at the family level seems to be a complex task that perhaps requires observing farther than the β1-α1 and β2-α2 loops that form part of the 2'-phosphate binding pocket. A positive charge in the first loop is not a feature in the other characterized NADP^+^-specific G6PDHs. Since groups I and II include dual, NADP^+^-preferring and NADP^+^-specific members, their conserved Arg in loop β2-α2 could participate in interactions with both NADP^+^ and NAD^+^. Therefore, discrimination against NAD^+^ seems to be a trait that has evolved more recently by different means. In the case of groups III and IV Asp seems to be frequent in the β2-α2 loop. Interestingly, a carboxylate is commonly observed at the pocket for the adenosine moiety in the dinucleotide-binding site of NAD^+^-dependent dehydrogenases [44], establishing hydrogen bonds with the hydroxyls of the ribose. Another major feature is that within group III His is more frequent in the R50 position. One might expect that a protonated histidine would provide a positive charge for 2'-phosphate binding. Also, His has shown to be able of π-stacking with the adenine base [45], but for this it must be neutral. Given that the protonation state of the histidine could vary within the pH range of the cytoplasm we remain cautious about assigning to a histidine the same role played by R50. Considering a potential role of His in the R50 position, it would be interesting to further investigate the effect of the pH of the environment in the specificity of enzymes in group III.

Two positive charges interacting with the 2'-phosphate are not uncommon when taking into account a more general survey of NADP^+^-binding proteins. By examining a non-redundant subset of the corresponding complexes in the PDB (S1 Text), we quantified that 35% of the structures present two positive residues establishing electrostatic interactions with the 2'-phosphate of NADP^+^. More than 50% of these cases involved Arg and Lys (S2 Text). But then again, kinetic data would be needed to establish whether this characteristic determines high selectivity for NADP^+^ over NAD^+^.

One illuminating example about how structural determinants of cofactor specificity could be associated to a specific motif in the cofactor binding pocket is the glutamate dehydrogenase family, whose members also possess a dinucleotide-binding Rossmann domain. The sequence similarity among NAD^+^-specific, NADP^+^-specific and dual representatives suggest that cofactor preference may have emerged multiple times in the course of evolution by using different structural strategies [46]. A structural motif located at the loop between one of the beta strands and one of the helices of the dinucleotide-binding domain contains a negatively charged residue whose function, according to the structural context is important to confer cofactor specificity. In the case of the NAD^+^-dependent clostridial enzyme, an Asp located in the structural motif binds the diol in the ribose-adenine of NAD^+^, but repels the negative charge of 2'-phosphate of NADP^+^ (together with bulky hydrophobic residues of the binding site offer no room to bind). In the case of the NADP^+^-dependent *E*. *coli* enzyme, the Asp in this position help to hold in place three positive charges that interact with the 2'-phosphate. In the case of the mammalian enzyme, dual specificity occurs because the negative charge in this position bridges a positive charge that provides a critical interaction with the 2'-phosphate of NADP^+^, and could also bind the diol of NAD^+^.

In order to increase the catalytic efficiency towards NAD^+^ from a NADP^+^-specific G6PDH exploring additional sites and mutations is necessary. In fact, it was expected that the K18T mutation might improve specificity towards NAD^+^, since this residue is observed in the dual *Lm*G6PDH. Notably, the specificity quotient for K18T was not different from the K18A mutant, which implies that if new interactions are established, they do not favor NAD^+^ more than NADP^+^ in the transition state of the reaction. Perhaps, evolving discrimination against NAD^+^ in *Ec*G6PDH involved loosing most of the interactions with the dinucleotide except for the high-energy ionic bridges with the 2'-phosphate moiety. Thus, improving the *E*. *coli* enzyme´s performance with NAD^+^ might require establishing a network of interactions beyond the single hydrogen bond that could be contributed by Thr at position 18.

## Conclusion

Homology modeling and MD simulations of *Ec*G6PDH in complex with NAD^+^ or NADP^+^ allowed us to identify K18 and R50 as major determinants of cofactor specificity. When the 2'-phosphate is present in the cofactor, R50 could establish electrostatic interactions with this moiety, further enabling π-stacking with the adenine ring. The energetic analysis of kinetic parameters of alanine mutants of these residues is in agreement with the *in silico* data of the NADP^+^ complex but the contribution of R50 to NAD^+^ binding was not reproduced by our conventional MD approach. The phylogenetic distribution of cofactor preferences in the G6PDH family indicates that enzymes with the same preference do not cluster together. The presence of two positive charges is not a common feature among the NADP^+^-specific G6PDHs, and sequence motifs in the β1-α1 and β2-α2 loops are important but not sufficient to determine cofactor specificity in the G6PDH family. We propose that discrimination against NAD^+^ could have evolved independently several times in the G6PDH family.

## Supporting Information

S1 Fig Kinetic parameters of G6P for mutant enzymes. K_M_ and *k*_cat_ in the table at the right were determined from the hyperbolic fit of the curves at the left side.
(TIF)

S1 Table Sequences selected for phylogenetic analysis. PDB and UniprotKB identifiers of the 31 sequences that were used to generate the Bayesian tree of Fig 6.
(XLSX)

S1 Text Database of non-redundant protein-NADP^+^ complexes. Starting with the total number of protein structures bound to NADP^+^ in the PDB, we selected a subset of representatives having less than 70% identity, showing a cofactor which is complete (48 atoms) and extended (more than 12 Å between carbon-2 in the Nicotinamide and carbon-6 in the Adenine). The PDB ID of each of the 330 structures matching these criteria is shown.
(PDF)

S2 Text Database of protein-NADP^+^ complexes possessing Arg and Lys interacting with the 2’-phosphate of NADP^+^. Starting from the database of the S1 Text, we filtered for structures possessing one Arg and one Lys, both closer than 4 Å from the 2’-phosphate of NADP^+^. The PDB ID of each of the 58 structures matching this criterion is shown.
(PDF)

## References

[1] Olavarría K, Valdés D, Cabrera R. The cofactor preference of glucose-6-phosphate dehydrogenase from *Escherichia coli*-modeling the physiological production of reduced cofactors. FEBS J. 2012;279: 2296–309. 10.1111/j.1742-4658.2012.08610.x 22519976

[2] Levy H. Glucose-6-Phosphate Dehydrogenases. 1979. 10.1002/9780470122938.ch3 367106

[3] Ragunathan S, Levy HR. Purification and characterization of the NAD-preferring glucose 6-phosphate dehydrogenase from *Acetobacterhansenii* (*Acetobacterxylinum*). Arch BiochemBiophys. 1994;310: 360–366. 10.1006/abbi.1994.1179 8179320

[4] Cornish-Bowden A. Fundamentals of Enzyme Kinetics. Fundamentals of Enzyme Kinetics. Elsevier; 1979.

[5] Dean AM, Golding GB. Protein engineering reveals ancient adaptive replacements in isocitrate dehydrogenase. ProcNatlAcadSci U S A. 1997;94: 3104–3109. 10.1073/pnas.94.7.3104 PMC20329 9096353

[6] Canback B, Andersson SGE, Kurland CG. The global phylogeny of glycolytic enzymes. ProcNatlAcadSci U S A. 2002;99: 6097–6102. 10.1073/pnas.082112499 PMC122908 11983902

[7] Kotaka M, Gover S, Vandeputte-Rutten L, Au SWN, Lam VMS, Adams MJ. Structural studies of glucose-6-phosphate and NADP+ binding to human glucose-6-phosphate dehydrogenase. Acta Crystallogr D Biol Crystallogr. 2005;61: 495–504. 10.1107/S0907444905002350 15858258

[8] Naylor CE, Gover S, Basak AK, Cosgrove MS, Levy HR, Adams MJ. NADP+ and NAD+ binding to the dual coenzyme specific enzyme *Leuconostoc mesenteroides* glucose 6-phosphate dehydrogenase: different interdomain hinge angles are seen in different binary and ternary complexes. Acta Crystallogr D Biol Crystallogr. 2001;57: 635–48. 11320304 10.1107/s0907444901003420

[9] Baugh L, Phan I, Begley DW, Clifton MC, Armour B, Dranow DM, et al Increasing the structural coverage of tuberculosis drug targets. Tuberculosis (Edinb). 2015;95: 142–8. 10.1016/j.tube.2014.12.003 25613812 PMC4361283

[10] Levy HR, Vought VE, Yin X, Adams MJ. Identification of an arginine residue in the dual coenzyme-specific glucose-6-phosphate dehydrogenase from *Leuconostoc mesenteroides* that plays a key role in binding NADP+ but not NAD+. Arch BiochemBiophys. 1996;326: 145–151. 10.1006/abbi.1996.0058 8579362

[11] Olavarria K, De Ingeniis J, Zielinski DC, Fuentealba M, Munoz R, McCloskey D, et al Metabolic impact of an NADH-producing glucose-6-phosphate dehydrogenase in *Escherichia coli*. Microbiology. 2014;160: 2780–2793. 25246670 10.1099/mic.0.082180-0

[12] Fersht A. Enzyme-substrate complementarity and the use of binding energy in catalysis In: Russel M, editor. Structure and Mechanism in Protein Science. New York: W. H. Freeman and Company; 1999 pp. 349–376.

[13] First EA, Fersht AR. Analysis of the role of the KMSKS loop in the catalytic mechanism of the tyrosyl-tRNAsynthetase using multimutant cycles. Biochemistry. 1995;34: 5030–5043. 7711024 10.1021/bi00015a014

[14] Serrano L, Horovitz A, Avron B, Bycroft M, Fersht AR. Estimating the contribution of engineered surface electrostatic interactions to protein stability by using double-mutant cycles. Biochemistry. 1990;29: 9343–9352. 10.1021/bi00492a006 2248951

[15] Zhao Y, Xiao J. Homology modeling and molecular dynamics simulation studies of human type 1 3-hydroxysteroid dehydrogenase: Toward the understanding of cofactor specificity. J Comput Chem. 2011;32: 33–42. 10.1002/jcc.21595 20607749

[16] Holmberg N, Ryde U, Bülow L. Redesign of the coenzyme specificity in L-lactate dehydrogenase from *Bacillus stearothermophilus* using site-directed mutagenesis and media engineering. Protein Eng. 1999;12: 851–856. 10556245 10.1093/protein/12.10.851

[17] Sali A, Blundell TL. Comparative protein modelling by satisfaction of spatial restraints. J Mol Biol. 1993;234: 779–815. 10.1006/jmbi.1993.1626 8254673

[18] Cole C, Barber JD, Barton GJ. The Jpred 3 secondary structure prediction server. Nucleic Acids Res. 2008;36: W197–201. 10.1093/nar/gkn238 18463136 PMC2447793

[19] Lüthy R, Bowie JU, Eisenberg D. Assessment of protein models with three-dimensional profiles. Nature. 1992;356: 83–5. 1538787 10.1038/356083a0

[20] Sippl M. Recognition of errors in three-dimensional structures of proteins. Proteins StructFunctBioinforma. 1993;17: 355–62. 10.1002/prot.340170404 8108378

[21] Phillips JC, Braun R, Wang W, Gumbart J, Tajkhorshid E, Villa E, et al Scalable molecular dynamics with NAMD. Journal of Computational Chemistry. 2005 pp. 1781–1802. 10.1002/jcc.20289 16222654 PMC2486339

[22] Lindorff-Larsen K, Piana S, Palmo K, Maragakis P, Klepeis JL, Dror RO, et al Improved side-chain torsion potentials for the Amber ff99SB protein force field. Proteins. 2010;78: 1950–8. 10.1002/prot.22711 20408171 PMC2970904

[23] Pearlman DA, Case DA, Caldwell JW, Ross WS, Cheatham TE, DeBolt S, et al AMBER, a package of computer programs for applying molecular mechanics, normal mode analysis, molecular dynamics and free energy calculations to simulate the structural and energetic properties of molecules. ComputPhysCommun. 1995;91: 1–41. 10.1016/0010-4655(95)00041-D

[24] Humphrey W, Dalke A, Schulten K. VMD: Visual molecular dynamics. J Mol Graph. 1996;14: 33–38. 10.1016/0263-7855(96)00018-5 8744570

[25] Thompson JD, Gibson TJ, Plewniak F, Jeanmougin F, Higgins DG. The CLUSTAL_X windows interface: flexible strategies for multiple sequence alignment aided by quality analysis tools. Nucleic Acids Res. 1997;25: 4876–82. 9396791 10.1093/nar/25.24.4876 PMC147148

[26] Ronquist F, Teslenko M, van der Mark P, Ayres DL, Darling A, Höhna S, et al MrBayes 3.2: efficient Bayesian phylogenetic inference and model choice across a large model space. Syst Biol. 2012;61: 539–42. 10.1093/sysbio/sys029 22357727 PMC3329765

[27] Lessmann D, Schimz KL, Kurz G. D-glucose-6-phosphate dehydrogenase (Entner-Doudoroff enzyme) from *Pseudomonas fluorescens*. Purification, properties and regulation. Eur J Biochem. 1975;59: 545–559. 1257 10.1111/j.1432-1033.1975.tb02481.x

[28] Anderson BM, Anderson CD. Purification and characterization of *Azotobacter vinelandii* glucose-6-phosphate dehydrogenase: dual coenzyme specificity. Arch Biochem Biophys. 1995; 10.1006/abbi.1995.1372 7639541

[29] Scopes RK, Testolin V, Stoter A, Griffiths-Smith K, Algar EM. Simultaneous purification and characterization of glucokinase, fructokinase and glucose-6-phosphate dehydrogenase from *Zymomonas mobilis*. Biochem J. 1985;228: 627–634. 2992451 10.1042/bj2280627 PMC1145031

[30] Haghighi B, Aghatabar AM, Shahsavari GH. Glucose 6-Phosphate dehydrogenase from *Streptomyces aureofaciens*: Ligand-induced conformational change. Iran J Sci Technol. 2005;29.

[31] Iyer RB, Wang J, Bachas LG. Cloning, expression, and characterization of the gsdA gene encoding thermophilic glucose-6-phosphate dehydrogenase from *Aquifex aeolicus*. Extremophiles. 2002;6: 283–289. 10.1007/s00792-001-0255-2 12215813

[32] Lessie TG, Wyk JC. Multiple forms of *Pseudomonas multivorans* glucose-6-phosphate and 6-phosphogluconate dehydrogenases: differences in size, pyridine nucleotide specificity, and susceptibility to inhibition by adenosine 5’-triphosphate. J Bacteriol. 1972. 10.1128/jb.110.3.1107-1117.1972 PMC247534 4402279

[33] Paraquat V, Ma J, Hager PW, Howell ML, Phibbs P V, Hassett DJ. Cloning and Characterization of the Pseudomonas aeruginosa *zwf* Gene Encoding Enzyme Important in Resistance to Methyl Cloning and Characterization of the *Pseudomonas aeruginosa zwf* Gene Encoding Glucose-6-Phosphate Dehydrogenase, an Enzyme Important in R. J Bacteriol. 1998;180: 1741–1749. 9537370 10.1128/jb.180.7.1741-1749.1998 PMC107085

[34] Steinbach RA, Sahm H, Schütte H. Purification and regulation of glucose-6-phosphate dehydrogenase from obligate methanol-utilizing bacterium *Methylomonas* M15. Eur J Biochem. 1978;87: 409–415. 668701 10.1111/j.1432-1033.1978.tb12390.x

[35] Rauch B, Pahlke J, Schweiger P, Deppenmeier U. Characterization of enzymes involved in the central metabolism of *Gluconobacter oxydans*. Appl Microbiol Biotechnol. 2010;88: 711–8. 10.1007/s00253-010-2779-9 20676631

[36] Cacciapuoti AF, Lessie TG. Characterization of the fatty acid-sensitive glucose 6-phosphate dehydrogenase from *Pseudomonas cepacia*. J Bacteriol. 1977;132: 555–563. 72065 10.1128/jb.132.2.555-563.1977 PMC221896

[37] Opheim D, Bernlohr RW. Purification and regulation of glucose-6-phosphate dehydrogenase from *Bacillus licheniformis*. J Bacteriol. 1973;116: 1150–1159. 4148096 10.1128/jb.116.3.1150-1159.1973 PMC246469

[38] Hansen T, Schlichting B, Schönheit P. Glucose-6-phosphate dehydrogenase from the hyperthermophilic bacterium *Thermotoga maritima*: Expression of the g6pd gene and characterization of an extremely thermophilic enzyme. FEMS Microbiol Lett. 2002;216: 249–253. 10.1016/S0378-1097(02)01021-2 12435510

[39] Vought V, Ciccone T, Davino MH, Fairbairn L, Lin Y, Cosgrove MS, et al Delineation of the roles of amino acids involved in the catalytic functions of Leuconostoc mesenteroides glucose6-phosphate dehydrogenase. Biochemistry. 2000;39: 15012–21. 11106479 10.1021/bi0014610

[40] Paton RS, Goodman JM. Hydrogen bonding and pi-stacking: how reliable are force fields? A critical evaluation of force field descriptions of nonbonded interactions. J ChemInf Model. American Chemical Society; 2009;49: 944–55. 10.1021/ci900009f 19309094

[41] Pletnev VZ, Weeks CM, Duax WL. Rational proteomics II: electrostatic nature of cofactor preference in the short-chain oxidoreductase (SCOR) enzyme family. Proteins. 2004;57: 294–301. 10.1002/prot.20205 15340916 PMC1476702

[42] Wendt UK, Hauschild R, Lange C, Pietersma M, Wenderoth I, von Schaewen A. Evidence for functional convergence of redox regulation in G6PDH isoforms of cyanobacteria and higher plants. Plant Mol Biol. 1999;40: 487–94. 10437832 10.1023/a:1006257230779

[43] Lee H-Y, Côté J-C. Phylogenetic analysis of gamma-proteobacteria inferred from nucleotide sequence comparisons of the house-keeping genes adk, aroE and gdh: comparisons with phylogeny inferred from 16S rRNA gene sequences. J Gen Appl Microbiol. 2006;52: 147–58. 16960331 10.2323/jgam.52.147

[44] Carugo O, Argos P. NADP-dependent enzymes. I: Conserved stereochemistry of cofactor binding. Proteins. 1997;28: 10–28. 9144787 10.1002/(sici)1097-0134(199705)28:1<10::aid-prot2>3.0.co;2-n

[45] Churchill CD, Wetmore SD. Noncovalent interactions involving histidine: the effect of charge on pi-pi stacking and T-shaped interactions with the DNA nucleobases. J Phys Chem B. 2009;113:16046–58. 10.1021/jp907887y 19904910

[46] Engel P. Glutamate Dehydrogenases: The Why and How of Coenzyme. Specificity. Neurochem Res. 2014;39: 426–432. 10.1007/s11064-013-1089-x 23761034

