# Supplementary figures and images for: Determinants of Cofactor Specificity for the Glucose-6-Phosphate Dehydrogenase from Escherichia coli: Simulation, Kinetics and Evolutionary Studies
Source: PLoS One. 2016 Mar 24;11(3):e0152403. doi: 10.1371/journal.pone.0152403 (PMC4807051; doi:10.1371/journal.pone.0152403)

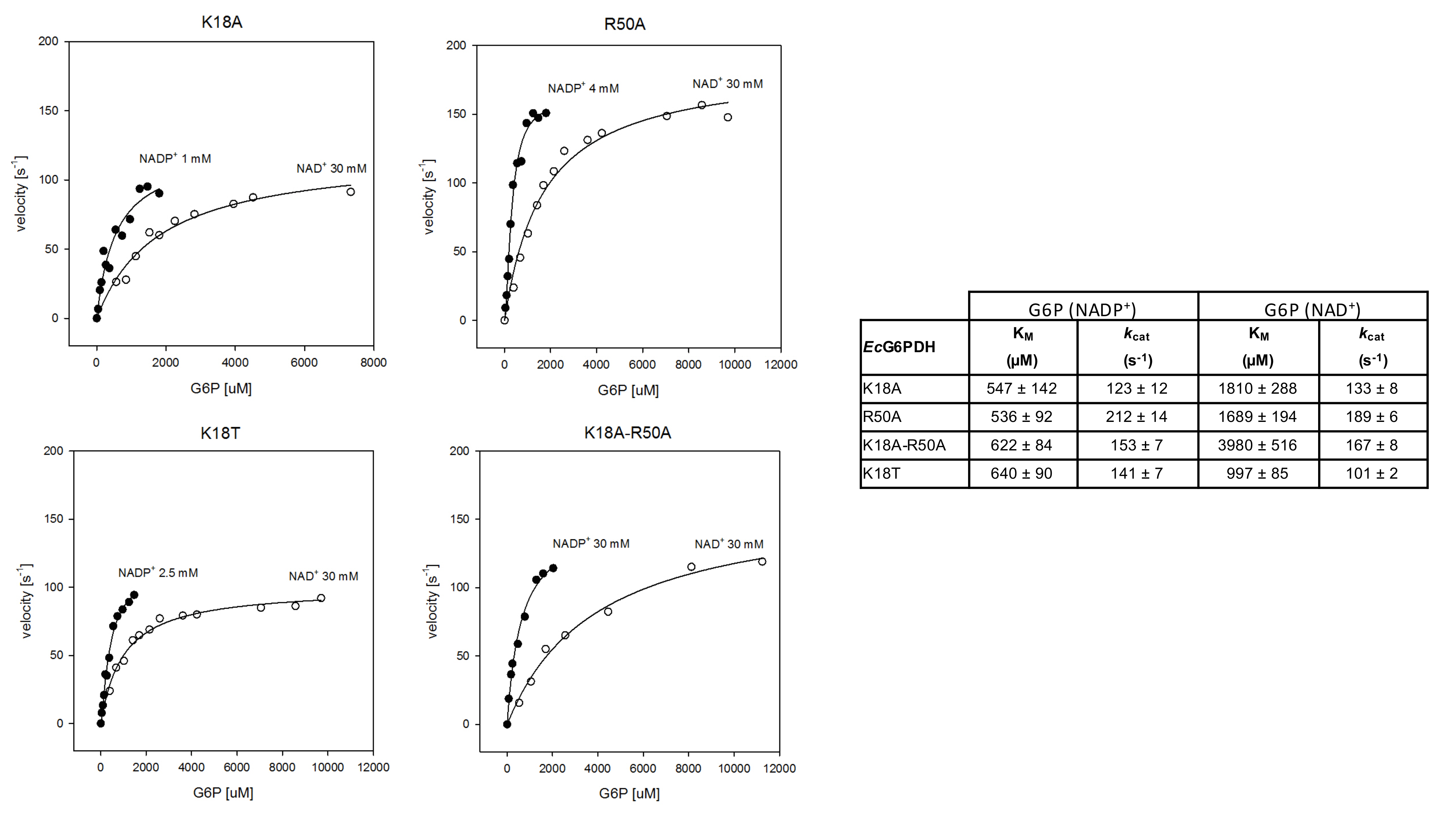

Supplement: S1 Fig — KM and kcat in the table at the right were determined from the hyperbolic fit of the curves at the left side. (TIF) [file pone.0152403.s001.tif]
