# Supplementary material for: Determinants of Cofactor Specificity for the Glucose-6-Phosphate Dehydrogenase from Escherichia coli: Simulation, Kinetics and Evolutionary Studies
Source: PLoS One. 2016 Mar 24;11(3):e0152403. doi: 10.1371/journal.pone.0152403 (PMC4807051; doi:10.1371/journal.pone.0152403)
Supplement: S1 Text — Starting with the total number of protein structures bound to NADP+ in the PDB, we selected a subset of representatives having less than 70% identity, showing a cofactor which is complete (48 atoms) and extended (more than 12 Å between carbon-2 in the Nicotinamide and carbon-6 in the Adenine). The PDB ID of each of the 330 structures matching these criteria is shown. (PDF) [file pone.0152403.s003.pdf]

1A27, 1A4I, 1AE1, 1AOE, 1C3V, 1CF2, 1CIV, 1CYD, 1D1G, 1D4O, 1DIH, 1DJL, 1DQA, 1E1M, 1E5Q, 1E7S, 1E7W, 1EDO, 1EQ2, 1EZ0, 1F06, 1G0O, 1GET, 1GIR, 1GQ2, 1GUF, 1GVE, 1H5Q, 1H6D, 1H7X, 1HDO, 1HYE, 1I36, 1IYZ, 1J3K, 1JA1, 1JA9, 1JAY, 1JUV, 1K0J, 1K8C, 1KC1, 1KEV, 1KMV, 1LQA, 1LUA, 1MXH, 1N7H, 1NVT, 1NYT, 1O2D, 1OAA, 1OG6, 1P0F, 1PGO, 1PNO, 1PQU, 1PS9, 1PZ0, 1PZ1, 1Q0Q, 1QFZ, 1QOR, 1QP8, 1QYV, 1RM4, 1RPN, 1RYD, 1S1P, 1SNY, 1T2A, 1TCS, 1TLL, 1U2G, 1US0, 1UXN, 1V3V, 1V9N, 1VBJ, 1VL8, 1VLJ, 1VP5, 1W6U, 1WMA, 1XG5, 1XHL, 1XKQ, 1XQ6, 1XU9, 1Y7T, 1YB5, 1YNQ, 1YQD, 1YS4, 1YVE, 1Z82, 1ZDL, 1ZGD, 1ZH8, 1ZK4, 2A87, 2AE2, 2AG8, 2AHR, 2AZN, 2B4Q, 2B5W, 2BD0, 2BGS, 2BH9, 2BKA, 2BL9, 2BSA, 2C0C, 2C29, 2CDC, 2CF6, 2CVZ, 2CWH, 2CY0, 2D2I, 2D5N, 2DBQ, 2EHQ, 2F1K, 2FR1, 2GCG, 2GGS, 2GN4, 2GQ2, 2GV8, 2H63, 2HK9, 2HXV, 2I3G, 2I76, 2IZ0, 2J8Z, 2JAH, 2JL1, 2O7P, 2O7S, 2Q0L, 2QE0, 2QHX, 2R6J, 2RAF, 2RCY, 2RHC, 2RIR, 2V6G, 2VN8, 2VQ3, 2VUU, 2W3W, 2WM3, 2WN6, 2WOW, 2WZW, 2X6T, 2X99, 2X9G, 2XVH, 2XW7, 2Y5D, 2YDX, 2YJZ, 2YLX, 2YUT, 2YW9, 2YYY, 2Z1M, 2ZAT, 2ZB4, 2ZCV, 2ZZC, 3A06, 3AFN, 3AI3, 3ASV, 3AU8, 3AXK, 3BAZ, 3BUV, 3C1O, 3CGE, 3CSE, 3D3F, 3D3W, 3DFR, 3DJJ, 3DTT, 3E8X, 3EAU, 3ETD, 3F8R, 3GQV, 3GWF, 3GY0, 3H2S, 3H4G, 3HJ3, 3HSK, 3HWR, 3I6I, 3IA4, 3ICC, 3IJP, 3ING, 3INV, 3IUP, 3IX9, 3JYN, 3JZ4, 3KBO, 3KGY, 3KRB, 3KVO, 3LNS, 3MJE, 3NGL, 3NRR, 3NX4, 3NZB, 3O03, 3O26, 3OID, 3OJF, 3OP4, 3P19, 3PDU, 3PEF, 3PHI, 3PYX, 3PZR, 3Q6J, 3QG2, 3QWB, 3QWF, 3RC1, 3RG9, 3RHL, 3RKU, 3S9U, 3SC6, 3SEF, 3SJ7, 3SJU, 3SLK, 3SQY, 3TOX, 3TQ8, 3TQH, 3TRI, 3TWO, 3U4C, 3UCE, 3UF0, 3UOY, 3W6Z, 3WBB, 3WCZ, 3WG6, 3ZBR, 3ZHB, 3ZQA, 4A03, 4A0S, 4AG3, 4ALK, 4AOS, 4AUB, 4B4O, 4B63, 4B7X, 4BMV, 4BVA, 4DMM, 4DPL, 4E5M, 4E5Y, 4EIL, 4EJ0, 4EJM, 4ESO, 4FC7, 4FDA, 4G5D, 4G5H, 4GCM, 4GI2, 4GIE, 4GMG, 4GVX, 4H4U, 4H8N, 4HA9, 4HFM, 4HNH, 4HP8, 4HXY, 4I5E, 4IDC, 4IIU, 4IJR, 4IMP, 4IMR, 4IQG, 4ITB, 4J1Q, 4J1T, 4J2O, 4JB1, 4JBI, 4JRO, 4K6F, 4K7Z, 4KJJ, 4KL9, 4KOA, 4KP7, 4KQW, 4KWH, 4L04, 4L4X, 4L8V, 4M7U, 4N5N, 4NHE, 4OL9
