# Supplementary material for: Determinants of Cofactor Specificity for the Glucose-6-Phosphate Dehydrogenase from Escherichia coli: Simulation, Kinetics and Evolutionary Studies
Source: PLoS One. 2016 Mar 24;11(3):e0152403. doi: 10.1371/journal.pone.0152403 (PMC4807051; doi:10.1371/journal.pone.0152403)
Supplement: S2 Text — Starting from the database of the S1 Text, we filtered for structures possessing one Arg and one Lys, both closer than 4 Å from the 2’-phosphate of NADP+. The PDB ID of each of the 58 structures matching this criterion is shown. (PDF) [file pone.0152403.s004.pdf]

1A27, 1AE1, 1CYD, 1D4O, 1DJL, 1EZ0, 1IYZ, 1JAY, 1LUA, 1NVT, 1OG6, 1PNO, 1PS9, 1QFZ, 1QOR, 1S1P, 1US0, 1V9N, 1VBJ, 1VP5, 1W6U, 1WMA, 2AZN, 2BD0, 2C29, 2GN4, 2HK9, 2WM3, 2YLX, 3BUV, 3D3W, 3H2S, 3H4G, 3IUP, 3JYN, 3KBO, 3KRB, 3KVO, 3LNS, 3O26, 3PEF, 3Q6J, 3QWB, 3SLK, 3TRI, 3W6Z, 3ZHB, 4A0S, 4ALK, 4EJM, 4G5D, 4G5H, 4GI2, 4GIE, 4IDC, 4J1T, 4J2O, 4L04
